# 0.7Pb(Mg_1/3_Nb_2/3_)O_3_-0.3PbTiO_3_ Phosphate Composites: Dielectric and Ferroelectric Properties

**DOI:** 10.3390/ma14175065

**Published:** 2021-09-04

**Authors:** Artyom Plyushch, Nerijus Mačiulis, Aliaksei Sokal, Robertas Grigalaitis, Jan Macutkevič, Alexander Kudlash, Natalia Apanasevich, Konstantin Lapko, Algirdas Selskis, Sergey A. Maksimenko, Polina Kuzhir, Juras Banys

**Affiliations:** 1 Faculty of Physics, Vilnius University, Sauletekio 9, LT-10222 Vilnius, Lithuania; nerijus.maciulis@ff.stud.vu.lt (N.M.); robertas.grigalaitis@ff.vu.lt (R.G.); jan.macutkevic@gmail.com (J.M.); juras.banys@ff.vu.lt (J.B.); 2 Institute for Nuclear Problems, Belarusian State University, 220006 Minsk, Belarus; sergey.maksimenko@gmail.com (S.A.M.); polina.kuzhir@uef.fi (P.K.); 3 Faculty of Chemistry, Belarusian State University, Nezalezhnastsi Ave. 4, 220030 Minsk, Belarus; sokolaa@bsu.by (A.S.); kudlash@bsu.by (A.K.); natalia_apanasevich@mail.ru (N.A.); LapkoKN@bsu.by (K.L.); 4 Department of Structural Analysis of Materials, Center for Physical Sciences and Technology, Sauletekio 3, LT-10257 Vilnius, Lithuania; algirdas.selskis@ftmc.lt; 5 Department of Physics and Matematics, Institute of Photonics, University of Eastern Finland, Yliopistokatu 7, FI-80101 Joensuu, Finland

**Keywords:** phosphates, PMN-PT, composites, dielectric properties, ferroelectric properties, densification

## Abstract

Composite materials with 83 wt.% of the 0.7Pb(Mg${}_{1/3}$Nb${}_{2/3}$)O${}_{3}$-0.3PbTiO${}_{3}$ distributed in phosphate-bonded ceramics were prepared at three different pressures. A phosphate matrix comprises a mixture of an aluminum phosphate binder and melted periclase, MgO. All samples demonstrate a homogeneous distribution of the ferroelectric perovskite phase and are thermally stable up to 900 K. At higher temperatures, the pyrochlore cubic phase forms. It has been found that the density of the composites non-monotonously depends on the pressure. The dielectric permittivity and losses substantially increase with the density of the samples. The fabricated composites demonstrate diffused ferroelectric–paraelectric transition and prominent piezoelectric properties.

## 1. Introduction

Composite materials filled with ferroelectric inclusions have received much attention in recent decades. Utilization of the different matrices and combinations with additional fillers allow to improve the properties of ferroelectrics and open wide perspectives for applications. In particular, composites with polymer matrices have potential applications in nanogeneration and energy harvesting [1,2,3,4], as well as ultrasonic transducers [5,6,7]. The addition of carbon nanotubes into such a system improves harvesting properties [8,9]. The composites comprising the mixture of ferroelectrics and ferromagnetics reveal the coupling of magnetic and electric polarizations [10,11]. The multiferroics have been widely used as sensors, transducers, memory elements, and spintronics [12,13,14,15]. Cement-based composites with ferroelectrics are used in structural health monitoring [16,17,18] and civil engineering fields [19,20].

Phosphate-bonded ceramics (PBCs) are intermediary materials between inorganic and polymer materials [21,22,23]. An important advantage of PBCs is the simplicity and low cost of the synthesis procedure. Phosphates are safe and eco-friendly. The preparation and curing steps occur at room temperature or with slight heating, without high-temperature sintering. Nowadays, PBCs can even be 3D printed [24]. After curing, the material demonstrates outstanding thermal stability and mechanical properties. Phosphates can act as a prospective host for a wide range of functional inclusions, which opens vast possibilities for biomedical [25,26], liquid wastes solidifying [27], drug delivery [24], and electromagnetic shielding applications [28,29]. Nevertheless, very few studies contribute to PBCs filled with ferroelectrics [30,31,32].

The only significant drawback of phosphate ceramics and cement for some utilizations is their relatively high porosity. However, this is a critical issue of ceramic materials in general. There are several approaches for densification, i.e., cold sintering process [33], microwave sintering [34], and increasing the pressure during material formation [35,36].

The present paper studied PBCs filled with a high concentration of ferroelectric inclusions. The composites were compressed under different pressures during the preparation procedure. The pressure-induced densification was studied for the case of chemically bonded material. Previously mentioned works study the densification of ceramics synthesized with sintering [35,36] that also impacts the density [37]. However, such densification is expected to apply to PBCs as well, despite the absence of sintering.

## 2. Materials and Methods

**Figure 1:**
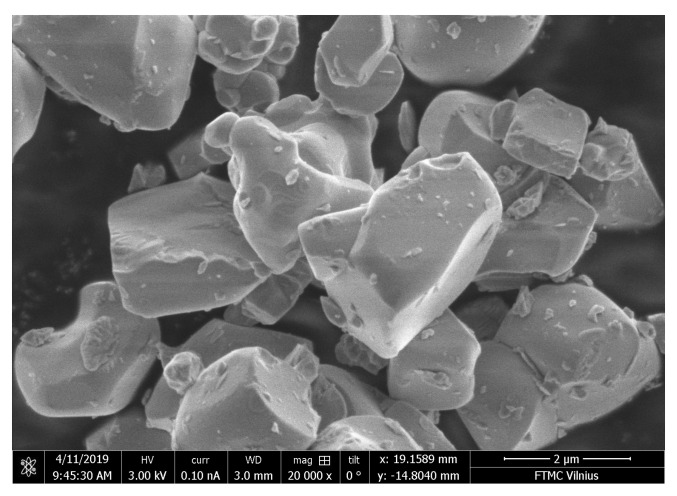
Scanning electron microscopy of the 0.7Pb(Mg${}_{1/3}$Nb${}_{2/3}$)O${}_{3}$-0.3PbTiO${}_{3}$ powder.

Ceramic composites consist of 3 components, i.e., a binder (Al(H${}_{2}$PO${}_{4}$)${}_{3}$), a filler (MgO), and a functional filler (0.7Pb(Mg${}_{1/3}$Nb${}_{2/3}$)O${}_{3}$-0.3PbTiO${}_{3}$). Commercially available by American Elements, lead magnesium niobate lead titanate (PMN-0.3PT, https://www.americanelements.com/printpdf/product/62022/datasheet, accessed on 3 September 2021) was used for the preparation of ceramic composites. The average grain size of the PMN-0.3PT powder is lower than 5 μm (see Figure 1). PMN-0.3PT is interesting as a filler for the composites due to its high dielectric permittivity, in combination with outstanding piezoelectric properties [38,39]. Commercially available by JSC Vostokogneupor (Russia, Yekaterinburg), melted periclase powder (MgO) was used as a filler. According to the supplier, the grain size of MgO is smaller than 63 μm.

The preparation procedure for the aluminum phosphate binder is as follows: Aluminum hydroxide powder Al(OH)${}_{3}$ was dispersed in distilled water. Then, the concentrated (85 wt.%) H${}_{3}$PO${}_{4}$ was added to aluminum hydroxide suspension. The content of water was calculated to obtain the final concentration of orthophosphoric acid of 60 wt.%. The mixture reacted under constant stirring and heating up to 373 K. The synthesis time takes approximately 60–90 min. The reagents interacted according to the equation: Al(OH)${}_{3}$ + 3H${}_{3}$PO${}_{4}\to$ Al(H${}_{2}$PO${}_{4}$)${}_{3}$ + 3H${}_{2}$O. After obtaining the transparent viscous dispersion, the mixture was cooled to room temperature and diluted with distilled water to a density of ρ = 1.42 g/cm${}^{3}$.

The binder (0.3 g), filler (0.12 g), and PMN-0.3PT (2.01 g) were mixed in an agate mortar for 10 min and uniaxially pressed into the tablets with a diameter of 10 mm under 3, 6, and 8 US-tons. The prepared PBC/PMN-0.3PT composite samples are marked as 3 t, 6 t, and 8 t, respectively. Then, the composites were kept for 24 h at ambient temperature (293 K) and thermally treated up to 573 K with a heating rate of 1 K/min to speed up the curing process. As a result, the samples with a high content of PMN-0.3PT (83 wt.%) were prepared.

The scanning electron microscopy (SEM) with energy dispersive X-ray analysis (EDX) was performed on Helios NanoLab 650 microscope (Thermofisher Scientific, Hillsboro, OR, USA). Dielectric properties of the developed composites in the frequency range of 20 Hz–1 MHz were measured using an LCR HP4284A (Hewlett-Packard, Palo Alto, CA, USA), and at the frequencies of 1 MHz–300 MHz, a coaxial dielectric spectrometer with a vector network analyzer Agilent 8714ET Santa Clara, CA, USA) was employed. For temperature measurements, a homemade furnace and a cryostat with liquid nitrogen were used. AixaCCT TF2000 analyzer (Aachen, Germany) equipped with a 4 kV supply was applied for piezoelectric measurements. The silver paste was used for electrodes. The density of the samples was evaluated by measuring the volume and mass of precisely cut samples with a parallelepiped shape. The thermal gravimetric analysis with scanning differential calorimetry (TGA/DSC) was performed using NETSCH STA 449 (Selb, Germany). Samples were tested in an ambient air atmosphere with a heating rate of 10 K/min. The powder X-ray diffraction (XRD) analysis was carried out on DRON 3.0 diffractometer (BOUREVESTNIK, JSC, Saint-Petersburg, Russia) using CoKα radiation (λ = 1.78896 Å), IDDC database PDF4+ was applied for identification. For XRD measurements of thermally treated samples, the following protocol was used: heating up to 473 K, 573 K (heating rate 1 K/min); 973 K, 1073 K, and 1273 K (heating rate 5 K/min), followed by 30 min of isothermal treatment.

## 3. Results and Discussion

**Figure 2:**
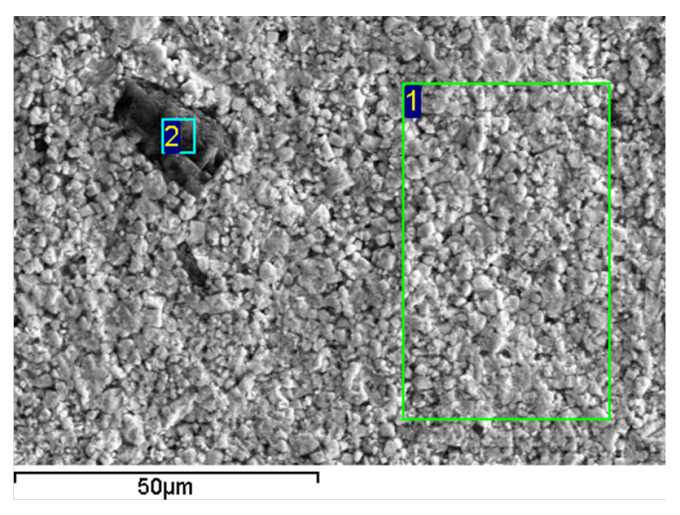
Scanning electron microscopy of the 8 t sample.

Scanning electron microscopy with energy dispersive X-ray analysis (Figure 2) of the 8 t sample reveals separate MgO grains surrounded with PMN-0.3PT grains (Table 1).

**Figure 3:**
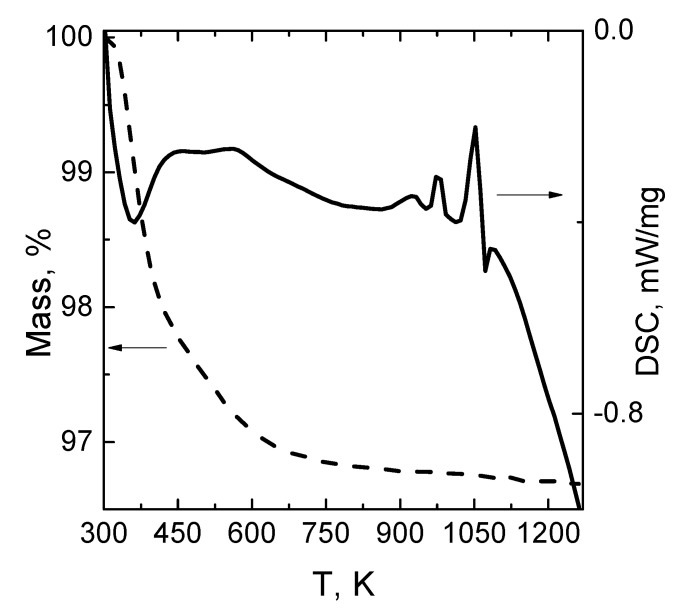
TG-DSC curves of the 8 t sample.

The result of TGA/DSC measurements of the 8 t composite material is presented in Figure 3. A total mass loss of approx. 3% occurs upon heating up to 750 K. In the temperature range of 300–450 K, the mass loss of 2%, accompanied by a sharp minimum in the DSC curve at 360 K, is associated with the absorbed water evaporation caused by the porous structure. The slight mass loss (approx. 1%) above 450 K occurs due to the evacuation of water obtained after the acid–base interaction processes in the PBC matrix. The series of peaks on the DSC curve at 900–1100 K without gravimetric effect is associated with high-temperature interactions between PMN-0.3PT and phosphate matrix. The developed composite remains stable up to 900 K.

**Figure 4:**
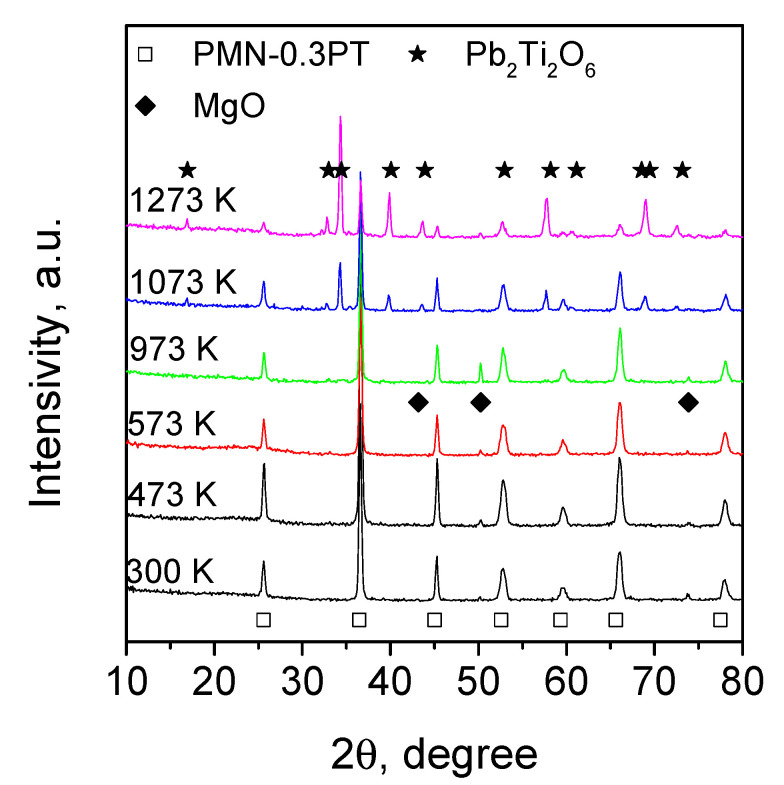
X-ray diffraction patterns of 8 t sample treated at different temperatures.

XRD analysis (Figure 4) of the as-synthesized material demonstrates the peaks from the perovskite PMN-0.3PT [01-088-1864] structure and small-intensity peaks from MgO [30-0794]. After high-temperature treatment, the intensity of the perovskite peaks gradually decreases. Additional reflections appear in the XRD spectra of samples treated at 1073 and 1273 K. These peaks are identified as pyrochlore Pb${}_{2}$Ti${}_{2}$O${}_{6}$ [26-142] cubic phase. Since both PMN-0.3PT and PBC are stable in the studied temperature range [29,40], the formation of pyrochlore phase is associated with the interaction between PBC and PMN-0.3PT at 900–1100 K (see Figure 3).

**Figure 5:**
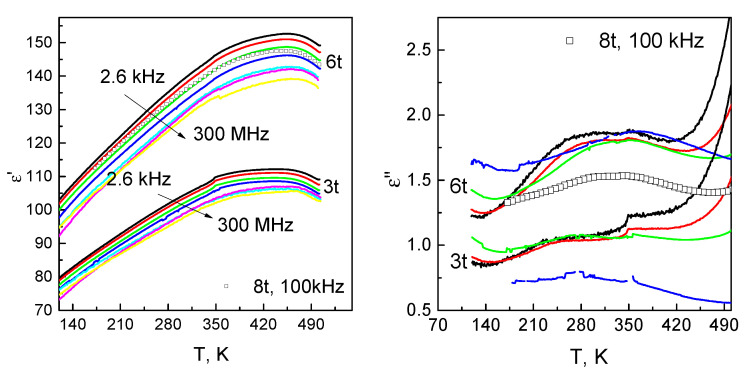
Real and imaginary parts of the dielectric permittivity as a function of temperature.

The temperature dependencies of the dielectric permittivity at different frequencies of 3 t and 6 t samples are presented in Figure 5. The results of the 8 t sample are close to 6 t, so only one frequency of 100 kHz is presented for comparison. The real part of $\varepsilon^{\prime }$ of the studied samples demonstrates a broad maximum near 460 K. The anomaly is related to the phase transition of 0.7Pb(Mg${}_{1/3}$Nb${}_{2/3}$)O${}_{3}$-0.3PbTiO${}_{3}$ from ferroelectric to paraelectric phase [41]. It is slightly different from the known peak position [41], which is probably due to possible defects of ferroelectric inclusions. Both samples demonstrate weak frequency dispersion of dielectric permittivity in the studied temperature range. The temperature corresponding to the maximum of $\varepsilon^{\prime }$ is frequency independent. Dielectric losses of the samples are not higher than 2 at temperatures up to 420 K. Upon further heating, the $\varepsilon^{\prime \prime }$ increases up to 2.5, and the strong frequency dispersion appears (Figure 5). This may indicate the Maxwell–Wagner relaxation at higher temperatures [42].

The densification effect is presented in Table 2. Several empirical models describe the densification behavior for conventional ceramics [35,36,43,44]. According to the mentioned literature, ceramics’ density monotonously increases with pressure. In the studied case, an increase in density is observed up to 680 MPa, which is followed by saturation at 680–904 MPa.

The effective media approach links the porosity of ceramics and ε [45]. This makes dielectric permittivity a sensitive tool to verify density measurements [46]. Similar to the density, ε demonstrates a plateau for samples pressed at 680–904 MPa.

Several factors influence the density of ceramics. The increase in pressure better compacts the powder and improves the density. The densification process is limited and saturates at higher pressures when the theoretical density is achieved [36]. At the same time, high pressure introduces the elastic strain. The cracks may appear as a result of mechanical strain release [47]. The sintering step also impacts the density of the ceramics [37] due to the grain growth [48,49] and the healing of the cracks [50]. For PBCs, the saturation of density at the pressures of 680–904 MPa may indicate the achievement of theoretical density. Additionally, the absence of the sintering step somehow limits the relative densities of the ceramics.

**Figure 6:**
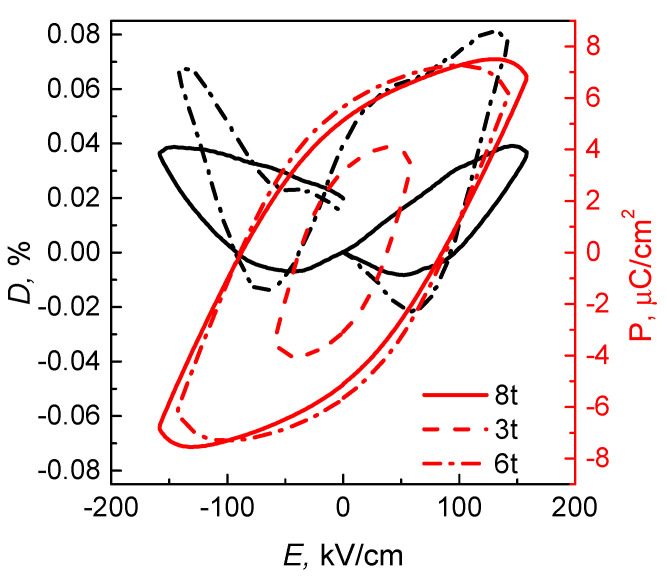
The strain and P-E hysteresis loops.

The electromechanical properties and hysteresis loop of the samples were measured at the frequency of 10 Hz at room temperature (Figure 6). The shapes of the displacements at the maximal electric field are not sharp, compared to the pure PMN-0.3PT ceramics [38]. This difference might relate to the impact of the PBC on the overall elastic properties of the composite. The P-E loops for 8 t and 6 t samples are close to each other with similar values of remnant polarisations and coercive fields. The rounded shape of the P-E loops demonstrates the dilution of ferroelectric properties. Such shape of the P-E loop is not typical for the PMN-PT conventional ceramics [46]; however, it was previously reported for the PMN-PT-based composites [16]. Similarly, the difference might be attributed to the composite nature of the samples. The PBC matrix introduces additional losses to the system.

## 4. Conclusions

In contrast to polymer-based [1,2,3] or cement-based [17,18] composites, the usage of phosphate matrices allows researchers to successfully synthesize composite materials with substantially higher (83 wt.%) content of ferroelectrics. Obtained composites demonstrate a homogeneous distribution of PMN-0.3PT grains with inclusions of MgO grains. Thermal stability is another important feature of the presented materials. Polymer matrices are thermally degradable at comparatively low temperatures [51,52]. The cement also loses mechanical strength at high temperatures [53], and therefore, modified compounds are required [54]. In contrast, phosphate-bonded ceramics gain mechanical properties after thermal treatment [29]. The composites are stable up to 900 K, while the pyrochlore Pb${}_{2}$Ti${}_{2}$O${}_{6}$ phase forms at a higher temperature.

The dependence of ceramic composites’ density on the applied pressure was studied. Densification was observed upon the increase in the pressure from 340 to 680 MPa. The increase in density was verified with the measurements of dielectric permittivity. The densest composite has 1.5 times higher $\varepsilon^{\prime }$ than that of the least dense composite. The temperature dependence of ε was studied in a wide range of 20 Hz–300 MHz. The $\varepsilon^{\prime }$ shows the maximum at the temperature of 460 K, which is attributed to the ferroelectric—paraelectric phase transition. The increase in dielectric losses at higher temperatures indicates the onset of the Maxwell–Wagner relaxation. The 8 t sample demonstrates a high piezoelectric strain of 0.04%. The shape of the D-E dependence is not sharp in comparison with the conventional ferroelectrics. That is attributed to different elastic properties of the PMN-0.3PT and phosphates.

To summarize, we fabricated a prospective material for a wide range of applications that combines the advantages of both PBCs and ferroelectrics. The preparation is simple and environmentally friendly. The absence of the sintering step allows loading the samples with additional fillers such as carbon allotropes or ferromagnetics, but more importantly, avoiding interaction between them. Additionally, it allows dodging the evacuation of lead from lead-based ferroelectrics, which is extremely important due to the RoHS directive.

## Figures and Tables

**Table 1:** Elemental content of the areas in Figure 2.

Area	O	Mg	Al	P	Ti	Nb	Pb
1	71.26	4.41	1	2.44	3.73	6.39	10.77
2	53.68	46.14	0.1	0.02	0.02	0.02	0.03

**Table 2:** Density of the samples as a function of the applied pressure.

Applied Pressure, MPa	340	680	904
Density, g/cm${}^{3}$	5.48	6.10	5.94
ε, at 100 kHz, 450 K	109.5-1.05*i*	148.67-1.68*i*	147.56-1.41*i*

## Data Availability

Data is contained within the article.
